# Visceral adipose tissue and residual cardiovascular risk: a pathological link and new therapeutic options

**DOI:** 10.3389/fcvm.2023.1187735

**Published:** 2023-07-27

**Authors:** Arturo Cesaro, Gianantonio De Michele, Fabio Fimiani, Vincenzo Acerbo, Gianmaria Scherillo, Giovanni Signore, Francesco Paolo Rotolo, Francesco Scialla, Giuseppe Raucci, Domenico Panico, Felice Gragnano, Elisabetta Moscarella, Olga Scudiero, Cristina Mennitti, Paolo Calabrò

**Affiliations:** ^1^ Department of Translational Medical Sciences, University of Campania “Luigi Vanvitelli”, Naples, Italy; ^2^ Division of Cardiology, A.O.R.N. “Sant'Anna e San Sebastiano”, Caserta, Italy; ^3^ Unit of Inherited and Rare Cardiovascular Diseases, A.O.R.N. Dei Colli “V. Monaldi”, Naples, Italy; ^4^ Department of Molecular Medicine and Medical Biotechnology, University of Naples Federico II, Naples, Italy; ^5^ Ceinge Biotecnologie Avanzate Franco Salvatore S. C. a R. L., Naples, Italy; ^6^ Task Force on Microbiome Studies, University of Naples Federico II, Naples, Italy

**Keywords:** obesity, visceral adipose tissue, residual cardiovascular risk, GLP-1 agonists, liraglutide, semaglutide

## Abstract

Obesity is a heterogeneous disease that affects almost one-third of the global population. A clear association has been established between obesity and cardiovascular disease (CVD). However, CVD risk is known to be related more to the local distribution of fat than to total body fat. Visceral adipose tissue (VAT) in particular has a high impact on CVD risk. This manuscript reviews the role of VAT in residual CV risk and the available therapeutic strategies for decreasing residual CV risk related to VAT accumulation. Among the many pathways involved in residual CV risk, obesity and particularly VAT accumulation play a major role by generating low-grade systemic inflammation, which in turn has a high prognostic impact on all-cause mortality and myocardial infarction. In recent years, many therapeutic approaches have been developed to reduce body weight. Orlistat was shown to reduce both weight and VAT but has low tolerability and many drug-drug interactions. Naltrexone-bupropion combination lowers body weight but has frequent side effects and is contraindicated in patients with uncontrolled hypertension. Liraglutide and semaglutide, glucagon-like peptide 1 (GLP-1) agonists, are the latest drugs approved for the treatment of obesity, and both have been shown to induce significant body weight loss. Liraglutide, semaglutide and other GLP-1 agonists also showed a positive effect on CV outcomes in diabetic patients. In addition, liraglutide showed to specifically reduce VAT and inflammatory biomarkers in obese patients without diabetes. GLP-1 agonists are promising compounds to limit inflammation in human visceral adipocytes.

## Introduction

Obesity is a heterogeneous disease that affects almost one-third of the world's population (1). Kelly et al. showed that if there is no change in this trend by 2030, 57.8% of the world's population will be overweight or obese (2). Consequently, obesity can be considered a pandemic (3). Obesity is a disease that affects all age groups. The prevalence of obesity is higher in women of any age and older people (4). Moreover, obesity in adolescence, in recent years, has become a global health emergency with increased cardiovascular disease (CVD) risk in adulthood (5).

Probably the most critical risk factors for developing obesity are the adoption of a sedentary lifestyle and a fat-rich diet (6). The increased availability of more nutrient-dense foods (with more critical marketing in recent years) could explain the weight gain among populations (7). Changes in food culture are considered the primary driver of the increased prevalence. Conversely, changes in lifestyle and less physical activity are cofactors of this expansion (7).

According to the WHO's BMI classification criteria, *overweight* is defined as a BMI > 25 kg/m^2^ and obesity as a BMI > 30 kg/m^2^ (8). The large inter-individual variability in the percent of fat according to age, sex, or ethnicity illustrated the lack of sensibility of this criterion (9, 10). Hence, in 2004, WHO experts proposed lower BMI cutoffs for the diagnosis of obesity for Asian people (11). Furthermore, BMI does not correlate precisely with the prevalence of metabolic abnormalities among ethnic groups. Indeed, Caucasian people have an equivalent prevalence of metabolic abnormalities to other ethnic groups with higher BMI (12).

Epidemiologic studies have depicted the relationship between obesity and 20 health outcomes (4). A strong association is clear with CVD, such as ischemic heart disease, ischemic and hemorrhagic stroke, and hypertensive heart disease, as well as with conditions that could be risk factors for impaired cardiac function, such as chronic kidney disease (CKD) or diabetes mellitus (DM) (4). About 30% of deaths worldwide are due to CVD; nowadays, CVD is the primary cause of mortality and early mobility globally (13).

The detrimental effects of obesity on cardiovascular health are well-known and mediated through intermediate conditions, like diabetes mellitus, dyslipidemia, and hypertension (13). Conversely an increased muscle mass is associated with better insulin sensitivity and glucose metabolism, lower rates of type 2 diabetes mellitus and cardiovascular events (14).

The inability to discriminate between fat mass and fat free mass by BMI may lead to erroneous assumptions about cardiovascular risk: the so-called “obesity paradox”. Lean subjects with preserved muscle mass are at lower cardiovascular risk than obese subjects (15).

For years, it has been known that CVD risk is related more to the local distribution of fat than to total body fat. There are anatomical, cellular, molecular, metabolic, clinical, and prognostic distinctions between visceral adipose tissue (VAT) and subcutaneous adipose tissue (SAT). The first has a high weight in CVD risk (16). Consequently, this manuscript aims to convey the available therapeutic strategies for clinicians to decrease the residual CVD risk related to VAT accumulation.

## Subcutaneous and ectopic adipose tissue: what are the differences?

Obesity is a heterogeneous disease related to adipose tissue (AT) accumulation. In women, the accumulation of AT is maximal in the hips and thighs, whereas in men it tends to accumulate in the trunk and upper body (16, 17). It depends mainly on the influence of hormones, especially estrogen, and on the activity of lipoprotein lipase (LPL, which allows TG accumulation in AT), which is more intense for women in the gluteal region. The literature describes two types of adipose tissue: subcutaneous (SAT) and ectopic (EAT) adipose tissue. There are different EAT types, including VAT (18).

Regarding total AT, 80%–90% is subcutaneous (16, 19); that is, it tends to form beneath the skin. It is divided into superficial and deep layers. The latter grows when obesity develops and correlates more strongly with CVD and obesity-related insulin resistance (20). Pubertal and post-pubertal women especially accumulate SAT. However, in postmenopausal women, the percentage of VAT increases (21). SAT works as a metabolic sink with the accumulation of TG in adipocytes, and it grows with surplus energy intake (i.e., a high-calorie diet). The most frequent locations of SAT storage are the abdominal, femoral–gluteal, and back (16). Abdominal AT is divided into subcutaneous and intra-abdominal (16). When the ability of SAT to store AT is exceeded (or impaired), AT starts to accumulate in locations that generally are not usual for adipose tissue storage, forming the EAT (22).

Visceral adipose tissue is classically ectopic, because lipid accumulation occurs in normally lean tissues such as the liver, the heart (pericardial, epicardial, and intramyocardial), and skeletal muscle or in areas of the body usually not associated with adipose tissue storage and containing only small amounts of fat (renal sinus, pancreas, thoracic, periaortic, perivascular) (18, 22, 23). Even within the abdominal superficial adipose tissue, the deep adiposity exhibits a metabolic phenotype closer to that of VAT (24). Any ectopic fat may be a “dysfunctional” adipose tissue, even if the debate is unresolved about ectopic fat as a marker or mediator of cardiometabolic diseases (23, 24).

## Liver and pericardial VAT

Ectopic fat deposits are VAT, intramuscular fat, fatty liver, perivascular and pericardial fat, myocardial steatosis, and renal situs fat. They may be subdivided into those causing systemic or local metabolic effects (18). These effects explain the relationship between EAT accumulation and CVD. Some of the most crucial systemic effects of EAT are the development of a low-grade systemic inflammatory status and/or insulin resistance (22). The contribution of VAT to their development is greater than fatty-liver or intra-muscular fat (indeed, they have smaller volume) (18). Nevertheless, a strong relationship between nonalcoholic steatohepatitis (NASH) and metabolic syndrome (MS) is known (25). Non-alcoholic fatty liver disease (NAFLD) is explicated as the liver's storage of lipids (especially TGs). The liver's fat content could be quantified with ultrasonography but is more accurate with magnetic resonance imaging (MRI) or computed tomography (CT) (26). NASH may reveal the risk of developing DM II and CVD. Indeed, this condition is characterized by the greater release of very low-density lipoproteins (VLDLs) and, hence, the heightened concentration of Apo-B lipoproteins (27), the development of impaired insulin sensitivity (28), and the release of inflammatory factors (29). Several studies have displayed that NASH could provoke coronary atherosclerosis and narrowing carotid arteries (29).

The relationship between adipose tissue storage and the heart could have two possible implications. One is the storage around the heart between the myocardium and pericardium (epicardial adipose tissue), and the other is the potential storage of AT into cardiomyocytes (myocardial steatosis) (30). Under physiological conditions, epicardial adipose tissue has an important function for the myocardium as thermogenic, mechanic, or metabolic support (30). Furthermore, perivascular fat has vasoactive properties (31). This valuable work of the perivascular/epicardial adipose tissue becomes dysfunctional when obesity develops (32). Indeed, excess EAT around the heart causes hypertrophy of the myocardial tissue, fibrosis, and the reduction of the synthesis of adiponectin through the heightened release of inflammatory factors (32, 33). The Framingham Heart Study clarifies the role of epicardial AT as an independent cardiovascular (CV) risk factor (34). Wang et al. also showed the relationship between this AT and components of MS as DM II, decreased HDL-C levels, hypertension, coronary disease, greater TG levels, and VAT (35). Moreover, several recent studies conveyed the positive relationship between the thickening of the pericardial AT and the prevalence of atrial fibrillation (33).

Conversely, myocardial steatosis (storage of lipids in cardiomyocytes) contributes, with local effects, to cardiac dysfunction. More lipids accumulate in cardiomyocytes in the hearts of patients with DM II or impaired glucose intolerance (36).

Another perivascular location of EAT is the accumulation of lipids around renal arteries (renal situs fat). It is associated with poor control of hypertension and chronic kidney disease (37, 38).

## Main pathomechanisms of VAT

The reasons for the correlations between VAT and clinical diseases are unknown. The most recognizable adverse effects of the accumulation of VAT are the development of metabolic alterations, insulin resistance and systemic inflammatory status (39).

### Free fatty acids

Anatomically, a more significant number of large and dysfunctional adipocytes form VAT. All types of visceral fat are metabolically active. They are insulin-resistant (few insulin receptors), hyper-lipolytic, and resistant to the anti-lipolytic effect of insulin, so they favor the accumulation of AT in ectopic tissues (40, 41). The high lipolytic activity of the abdominal VAT constantly releases free fatty acids (FFAs), drained through the portal vein, so FFAs accumulate in the liver.

This accumulation of FFAs and glycerol in the liver leads to alterations in the lipid and glucose metabolism. The liver reduces the binding of the insulin and increases the content of lipids and the secretion of lipoproteins rich in triglycerides (VLDL, Apo-B) (42, 43). Increased production of glucose and hyperinsulinemia, could explain the relationship between VAT and DM II (43).

Metabolically, VAT has a greater density of glucocorticoid (44) and androgen receptors (44). Subsequently, in men after 50 years, VAT tends to increase (4). By contrast, the binding capacity of estrogens is greater in SAT (45). In women, VAT tends to increase in adulthood (44). In addition, VAT is more sensitive to catecholamine-induced lipolysis (46). More lipolysis and higher levels of FFAs lead to hyperglycemia, hyperinsulinemia, and hypertriglyceridemia by propagation of insulin resistance to the liver.

### Adipokines

VAT is regarded as endocrine system secreting several adipokines, e.g., leptin, adiponectin, omentin, visfatin, resistin, and apelin (47). Adipokines are mediators of body composition, due to their pleitropic effects on metabolism. In adiposity, especially in VAT, production of adipokines are altered.

Leptin is involved in the regulation of body weight and fat distribution (48). In overweight and obesity hyperleptinemia and resistance to leptin increase, resulting in reduced energy expenditure, hyperinsulinemia and hyperlipidemia (49). The greater the VAT, the higher the blood levels of leptin, a marker of MS that predicts CV risk) (45, 46, 50, 51).

Adiponectin is a hormone which plasma levels decrease with visceral fat accumulation, although it is secreted only by adipose tissue (52). By reducing the levels of circulating adiponectin, the stimulation of fatty acid oxidation and glucose uptake in skeletal muscle are reduced, worsening whole-body energy homeostasis (53). Also in the liver fatty acid oxidation is blunted, together with inhibition of glucose production (53). Therefore obesity-associated hypoadiponectinemia may contribute to hepatic steatosis. Adiponectin exemplifies the less production of protective adipokines (23).

Omentin enhances insulin action by stimulating insulin-mediated glucose uptake, without effect on basal glucose transport (54). Serum omentin level is significantly lower in overweight subjects (55). Other adipokines that lost their protective effects are vaspin (inhibition of reactive oxygen species) and apelin (enhancement of cholesterol efflux) (47). In contrast, adipokines that accentuate their detriment effects include resistin (interference with insulin action) and visfatin (insulin-mimetic actions) (56).

Overall, adipokines have different origins (adipocyte, stromal cells and type of VAT), mechanisms of action (direct, mediated, positive or negative) and effects on various metabolic circuits (56). Upstream, adipokine dysregulation due to adipose tissue dysfunction contributes to obesity-related metabolic diseases.

### Inflammation

Expansion of visceral adipose tissue depots is accompanied by inflammation (57). One of the first step is infiltration of new macrophages, which increase up to 10 times (23, 58). Adypocytes secrete high levels of monocyte chemotactic protein-1 (MCP-1), a potent chemiotactic chemokine promoting monocytes/macrophages accumulation (59). Also the macrophages derived from monocytes produce additional MCP-1 because they switch to a pro-inflammatory phenotype (60). Also the increase of production of TNFα reflects the influx of inflammatory cells within expending adipose tissue (61). It has long been known that obesity is associated with elevated circulating levels of IL-6 released from visceral fat, providing a strong link with inflammation (62). Visceral adipose tissue released greater amounts of IL-6 compared with abdominal subcutaneous tissue (63).

Local inflammation is further promoted by immune cells resident in adipose tissue. Various types of T cells (CD4+, CD8+, natural killer) enrich VAT and produce interferon-γ (IFNγ). INFγ stimulates the differentiation of monocytes in activated macrophages (64).

Local VAT inflammation progresses to low-grade systemic inflammation, that is characterized as mildly elevated levels of circulating cytokines, chemokines, and acute phase reactants (61).

These signals from dysfunctional adipose tissue are primary stimuli for hepatic macrophages (Kupffer cells). Polarization to inflammatory phenotype of Kupffer cells propagates inflammation to liver (65). Furthermore, the liver starts to produce inflammatory mediators (41).

The release of interleukin-6 (IL-6) and tumor necrosis factor-alfa (TNF-alfa), with a proatherogenic and prodiabetic function, is higher in obese patients (16). Moreover, IL-6 is the primary mediator of CRP production in the liver, and blood levels of CRP will be increased in obese patients (16).

There is a close relationship between inflammatory and atherosclerotic disease (66). Visceral obesity is also related to hypercoagulability (67), peripheral arterial disease (68), atherosclerosis in carotid arteries (69, 70) and microalbuminuria (71).

### Insuline resistance

The release of FFA from dysfunctional adipocytes into the circulation is also diverted to muscle and other tissues (72). Decreased muscle glucose uptake and hepatic insulin resistance are other consequences of excess of FFA from visceral fat and can contribute metabolic complications, such as dyslipidemia and risk for type 2 diabetes (T2DM) (73). Fat accumulates ectopically pancreatic β-cells and occurs tipically with elevated levels of lipotoxic intermediates such as diacylglycerol (73). Lipotoxicity contributes to insulin impairment.

It has been reported that FFA levels were lower in metabolically healthy obesity subjects than those with metabolically unhealthy obesity (74), high level of FFA are associated with a higher incidence of T2DM and almost all patients with type 2 diabetes have fatty liver (75).

The association of visceral adiposity with dyslipidemia, inflammation, insulin resistance, glycemic abnormalities, hepatic steatosis and many other metabolic and vascular disturbances configure a picture of metabolic syndrome, contributing to increased atherosclerosis and cardiometabolic risk.

Insulin resistance, principally through the activation of the renin-angiotensin-aldosterone system and sympathetic activation, leads to hypertension (46). Obesity and visceral adiposity in particular account for most of the risk of hypertension (46, 50).

To show the role of VAT as an independent risk factor for metabolic and CV diseases, Ruiz-Castell et al. (60) divided a cohort of patients (*n* = 1,529) into quartiles based on estimated VAT according to Samouda's anthropometric measure (76). This trial showed that the odds ratio (OR) increased in parallel with the rise in VAT size. This relationship was more robust for men with hypertension (OR 11.83 vs. 8.21 for the fourth quartile), though the correlation was stronger for women than men for prediabetes and diabetes (OR = 7.57 vs. 5.41, for the fourth quartile), for hypercholesterolemia (OR = 5.28 vs. 2.26, for the fourth quartile), and for hypertriglyceridemia (OR = 14.62 vs. 6.78, for the fourth quartile).

## Visceral adipose tissue accumulation as a CV risk factor

VAT forms around the abdominal visceral organs, and constitutes about 5%–15% of the total AT (77). Several methods are available for measuring VAT. The most commonly used are anthropometric measures or bioelectrical impedance analysis (BIA) (78). However, these are inaccurate. Only CT and MRI could give a direct measurement of VAT, and the point for optimal image acquisition to estimate the VAT is 5–6 cm above the L4–L5 disk (78). A good estimation of VAT could also be obtained thanks to the development of dual-energy x-ray absorptiometry (DEXA) (23). It calculates VAT as the difference between total AT and SAT. In addition, ultrasound could help measure VAT by measuring the distance between the aorta and the internal face of the abdomen wall. Nonetheless, it is a method that depends strongly on the operator's skill (78). In real-world experience, these methods are reserved only for research scopes, so several anthropometric measures are created to allow an indirect measure of VAT. Perhaps the most used is waist circumference (WC), but the waist-to-hip ratio (WHR) and sagittal abdominal diameter are also available in clinical practice (78). If waist circumference represents VAT and SAT, hip circumference represents SAT only. Accordingly, a high WHR value may be significant for a large VAT (78). Nevertheless, a study showed that the most reliable surrogate for the measurement of VAT is WC (78). Several thresholds are accessible for WC. In the U.S., 102 cm in men and 88 cm in women are necessary for a diagnosis of MS (79). However, there may be ethnicity-specific differences in the WC threshold (80). Another anthropometric measure of VAT is neck circumference, but there is no solid literature on the topic (23). Samouda et al. described a new anthropometric measure for estimating VAT based on thigh and waist circumference and adjusted for BMI and age (43). It is: (6 × waist circumference −4.41 × proximal thigh circumference +1.19 × age −213.65) for men and (2.15 × waist circumference −3.63 × proximal thigh circumference +1.46 × age +6.22 × BMI −92.713) for women (76).

Ectopic fat localized around abdominal visceral organs is a significant risk factor for metabolic and CV disease, independent of total AT (19, 23).

## Visceral adipose tissue and residual cardiovascular risk

Residual CV risk comprises persistent CV risk; it is the possibility of incident vascular events or the progression of established vascular damage, despite optimized care (81). This could be associated with known CV risk factors not adequately treated with therapy or unknown CV risk factors (81, 82). This concept originated from the evidence of people experiencing new CV events or the recurrence of CV events despite evidence-based pharmacological therapy (61). Since the emergence of interventional trials, mainly statin trials, science has started to discuss residual CV risk (83).

**Figure 1:**
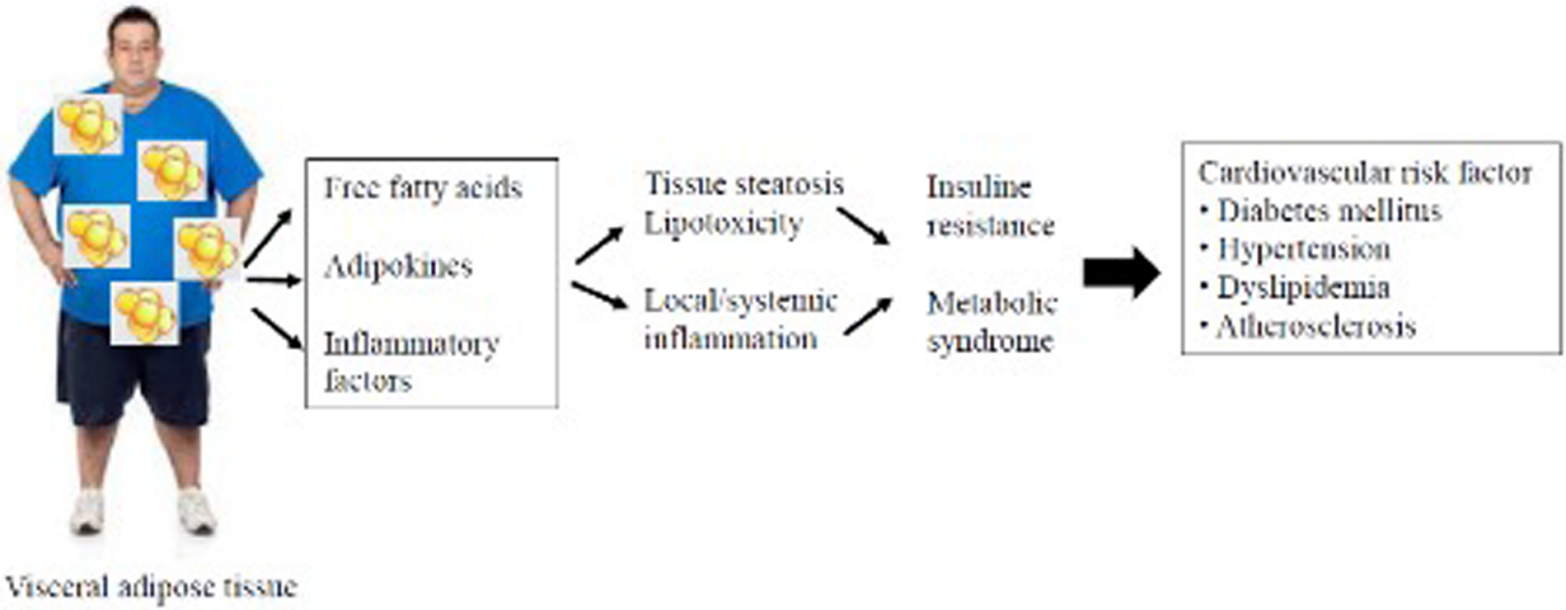
Schematic diagram from visceral adipose tissue to cardiovascular damage.

Many pathways could be involved in residual CV risk (Figure 1). The most important are lipidic, thrombotic, inflammatory, and diabetic patterns (84). Obesity, particularly the accumulation of VAT through the generation of low-grade systemic inflammation, increases the residual CV risk (77). The relationship between the development of atherosclerosis and inflammation has been known since studies demonstrated the presence of inflammatory cells, such as macrophages, in the arterial plaque and since activated T-lymphocytes were found at the site of plaque rupture or erosion (85). Furthermore, the risk of plaque rupture could depend more on the number of macrophages than on plaque size (86).

The prognostic value of inflammation in patients undergoing percutaneous coronary intervention (PCI) was evaluated by Kalman et al. among 7,000 patients (66). Subsequently, 38% of these patients had persistently high residual inflammatory risk (RIR; defined as high blood levels of high sensitive C-reactive protein ≥2 mg/L before and after >4 weeks after the PCI). All-cause mortality and myocardial infarction (MI) incidence rates are higher in these patients at the 1-year follow-up (87). Therapy targeting interleukin-1β with canakinumab at a dose of 150 mg/die proved in the CANTOS trial to reduce the incidence rate of the primary endpoint (composite of nonfatal MI, nonfatal stroke, or CV death). The incidence rate for events was 3.86 per 100 person-years in the 150 mg group, despite 4.50 per 100 persons in the placebo group, with a hazard ratio of 0.85 in the 150 mg group (*p* = 0.021) (88). Nowadays, no drugs are available that act selectively on RIR.

Furthermore, there is a known relationship between adiposity and dyslipidemia. Regarding the lipidic pathway, the most crucial part of residual CV risk is associated with abnormal triglyceride levels (TG)-rich lipoproteins (TGRLs), their remnants, or alterations in the number or function of HDL (high density lipoprotein) (89). Dyslipidemia associated with non-LDL (low-density lipoprotein) is called *atherogenic dyslipidemia* or *atherogenic dyslipoproteinemia* (90).

TGRLs derive from the diet (chylomicrons and remnants) and the liver (very low-density lipoprotein and their remnants) (59). The hydrolyzation of TGRLs by LPL transforms these lipoproteins into relatively cholesterol-rich molecules, termed *cholesterol remnants* and measured with the formula: total cholesterol-HDL-C—LDL-C (89, 90).

Even in patients well-treated for hypertension, some data support the persistence of residual CV risk, even with an optimal control of blood pressure (91–93).

## Pharmacological strategies against visceral adipose tissue accumulation

In recent years, many therapeutic approaches have been developed to reduce body weight.

Molecules of novel therapeutic classes have emerged, such as tirzepatide, the first dual glucose-dependent insulinotropic polypeptide/glucagon-like peptide-1 receptor agonist (GIP)/GLP-1 RA), which has recently demonstrated a body weight reduction exceeding 20% in people with obesity, coupled with improved cardiometabolic measures (94). Extensively studied for weight management is lorcaserin, a selective 5-HT2C receptor agonist (95). Also the insulin sensitizers thiazolidinediones such as pioglitazone have shown in combination with simvastatin to reduce adipose tissue inflammation in subjects with MS (96).

There are five Food and Drug Administration (FDA)-approved medications for managing body weight (97). A summary of the main drugs and their characteristics is provided in Table 1.

**Table 1:** Characteristics comparison of the principal drugs for the treatment of obesity.

Name	Orlistat (97, 98)	Phentermine-topiramate (97, 99)	Naltrexone-bupropion (97, 100)	Liraglutide (97, 99)	Semaglutide (97, 101)
Mechanism of action	Pancreatic and gastric lipase inhibitor	Sympathomimetic and gamma-aminobutyric acid (GABA) receptor agonist	Opioid receptor agonist- norepinephrine-dopamine reuptake inhibitor	Glucagon-Like Peptide 1 (GLP-1) agonist	Glucagon-Like Peptide 1 (GLP-1) agonist
Dosage	120 mg	From 3.75 mg/23 mg to 15 mg/92 mg	7.2 mg/78 mg	From 0.6 mg to 3 mg	From 0.25 mg to 2.4 mg
Route of administration	Oral	Oral	Oral	Subcutaneous	Subcutaneous
Frequency of administration	Three times daily: before, during, or immediately after every deal containing fat	Once a day	From once a day to two tablets twice a day	Once a day	Once a week
Principal side effects	Gastrointestinal adverse effects including steatorrhea	Nervous system side effects: • headaches • insomnia • paresthesia • irritability • dizziness • anxiety • depression	• nausea • constipation • dizzinessess • dry mouth • vomiting	Gastrointestinal side effects could improve through the days: • nausea • diarrhea • vomit • constipation • abdominal pain • dyspepsia	Gastrointestinal side effects could improve through the days: • nausea • diarrhea • vomit • constipation • abdominal • pain • dyspepsia

The first is orlistat, an oral drug shown to reduce body weight by 5% or more. It is a pancreatic and gastric lipase inhibitor (98). Several studies have shown the efficacy of orlistat. Khera et al. showed a reduction of 5% of body weight in 44% of patients treated with orlistat despite 23% of those in treatment with placebo and, after 1 year of follow-up, a more significant reduction of body weight (−2.6 kg) in patients receiving orlistat despite a placebo (102). Orlistat's tolerability is not optimal. Indeed, this drug may cause steatorrhea to impair the absorption of fat as well as some vitamins and nutrients (76). Special attention must be given to patients receiving levothyroxine, warfarin, antiepileptics, and antiretroviral medications (97). Orlistat may interfere with their function. Furthermore, it is contraindicated for pregnant and breastfeeding women, and there are no data about its use for children (97). In a 2011 multicenter study, orlistat 60 mg showed the ability to reduce VAT to a greater extent than placebo in patients with a BMI of 25–34.9 kg/m^2^ without DM (65). Indeed, after 24 weeks of treatment, people receiving orlistat with a low-calorie diet and physical exercise reached a median reduction of VAT of 15.7%, compared to 9.4% of people receiving a placebo. In addition, orlistat-treated patients reached a more significant total mass fat loss than placebo-treated patients (−4.65 vs. −3.01 kg, *p* < 0.05) (100).

Another drug in use against obesity is the combination of an opioid receptor antagonist (naltrexone) and a norepinephrine–dopamine reuptake inhibitor (bupropion) (103). In a meta-analysis 55% of patients receiving naltrexone–bupropion lowered their body weight by 5% compared to 23% in the placebo-treated population (104). After 1 year of follow-up in naltrexone–bupropion-treated patients, there was 5 kg excess weight loss compared to placebo (104). Problems with the tolerability of this drug are frequent. Mainly, it is contraindicated in patients with uncontrolled hypertension (105).

In a phase 2 trial naltrexone/bupropion obtained a reduction of VAT mass (−15.0%) greater than placebo (−4.6%) and proportional with weight loss in 80 obese subjects treated for 24 weeks (106).

In 2012, the FDA approved the commercialization of the association between phentermine–topiramate. The first is a sympathomimetic that causes an increase in blood and central nervous system levels of norepinephrine. The second is a gamma–aminobutyric (GABA) receptor agonist, also used for treating epilepsy or headaches (83). The fixed drug combination showed optimal results in reducing body weight, 4.7%–10.4% weight loss (99) and also improvements in waist circumference measures, suggesting possible diminution in visceral fat depots (107). However some questions about its long-term safety remain (97). The principal side effects concern the nervous system, such as depression, paresthesia, and insomnia (99, 107). Furthermore, it is contraindicated in pregnant women. Due to the lack of long-term safety (99), the European Medicines Agency (EMA) did not allow its commercialization.

Liraglutide and semaglutide are the last two drugs approved by the FDA and EMA to treat obesity (97, 108). They belong to the same antidiabetic drug family: the Glucagon-Like Peptide 1 (GLP-1) agonists (97). Between those, there are some distinctions in the efficacy of weight loss and in the frequency of subcutaneous administration.

The efficacy of weight loss with the daily administration of liraglutide 3.0 mg/daily was assessed in 2015 by the SCALE trial (109). A cohort of 3,731 obese patients (BMI > 30 or >27 kg/m^2^ with dyslipidemia or hypertension) were enrolled and divided into subjects receiving a daily dose of liraglutide 3 mg and subjects receiving a placebo as an adjunct to diet and exercise. After 56 weeks of follow-up, the liraglutide group achieved a mean loss of 8.4 ± 7.3 kg, and the placebo group lost a mean of 2.8 ± 6.5 kg (*p* < 0.001). Additionally, 63.2% of patients in the liraglutide group lost at least −5% of their body weight compared with 27.1% in the placebo group. A difference of 22.5% between the two groups was also seen for the rate of patients achieving −10% of body weight (109).

In 2022, Rubino et al. showed a more significant loss in body weight with semaglutide than with liraglutide in obese patients without diabetes (−15.8% vs. −6.4%, *p* < 0.01) with a higher rate of obtaining 10% or more weight loss (70.9% vs. 25.6%, *p* < 0.01). The rate of subjects discontinuing treatment was higher for liraglutide than semaglutide (27.6% vs. 13.5%) (110). Nevertheless, compared to placebo, semaglutide demonstrated a higher proportion of subjects discontinuing treatment due to side effects (especially gastrointestinal) (101, 111). Semaglutide is administered once a week; liraglutide is administered daily (97).

In 2016, liraglutide showed a positive role in CV outcomes in diabetic patients. In the LEADER trial, 9,340 diabetic patients with high CV risk were randomized to receive a placebo or liraglutide (112). The primary composite outcome was the first occurrence of death from CV causes, nonfatal myocardial infarction, or nonfatal stroke. After 3.8 years of follow-up, the primary outcome rate was 13% in the liraglutide group compared to 14.9% in the placebo group (*p* < 0.01 for noninferiority; *p* = 0.01 for superiority) (113).

In addition to liraglutide, semaglutide (114), dulaglutide (115) and efpeglenatide (116) in patients affected by DM II also showed the ability to reduce the rate of major adverse cardiovascular events (MACEs) compared to placebo.

In a *post-hoc* analysis of five trials, Davies et al. evaluated whether the CV risk increased during treatment with liraglutide despite comparators (placebo or orlistat) in a population both with and without DM (117). The primary composite outcome was the first occurrence of CV death, nonfatal myocardial infarction, or nonfatal stroke. Two of the five trials were adjudicated retrospectively. In liraglutide 3.0 mg, eight participants experienced adverse CV events, despite 10 participants in the comparators group. The hazard ratio (HR) for liraglutide 3.0 mg vs. its comparators was 0.42 (*p* = 0.07). For all liraglutide doses vs. all comparators, the HR increased to 0.50 (*p* = 0.11) (94).

Only with the ongoing Semaglutide Effects on Heart Disease and Stroke in Patients With Overweight or Obesity (SELECT) study (NCT03574597) will clinicians understand whether GLP-1 agonists (semaglutide) can reduce MACEs in obese patients with an established CV event (prior myocardial infarction, prior stroke, or peripheral arterial disease) without DM (118).

The once-daily administration of liraglutide 3.0 mg with physical exercise and a 500-kcal deficient diet, compared to a placebo over a median of 36 weeks on treatment, reduced the VAT measured with MRI (96). In a cohort of 128 obese patients (BMI > 30 or >27 kg/m^2^ with MS) without DM, in the liraglutide group there was a mean reduction in VAT of −12.49% compared with −1.63% in the placebo group (*p* < 0.0001). The liraglutide 3.0 mg group also obtained better results concerning secondary outcomes. Indeed, the estimated treatment disparities between the two groups were −33% for liver fat lost, −9.10% for abdominal SAT lost, −8.66% for lower body fatty tissue lost, and −8.64% for total body adipose tissue lost (119). Furthermore, in the liraglutide group, there was a reduction in CPR blood levels (−19.91%, *p* = 0.038) and in fasting blood glucose (−5.62%, *p* = 0.0048). In patients receiving liraglutide, the most frequent adverse events were gastrointestinal-related and upper respiratory tract infections but were low-grade episodes (grades 1–2). During the treatment, no patient had episodes of hypoglycemia (119).

Similar results came from Yu et al. (120). They enrolled a population of patients with a BMI > 28 kg/m^2^ with a prior diagnosis of DM II who had not undergone lifestyle interventions. They were divided into a study group (receiving liraglutide) and a control group (undergoing lifestyle interventions). After 12 weeks of follow-up, the liraglutide group obtained a more significant reduction of VTA calculated by the energy spectrum CT (−7.1 ± 10.17 cm^2^ in the study group vs. 0.91 ± 16.59 cm^2^ in the control group) (120).

**Figure 2:**
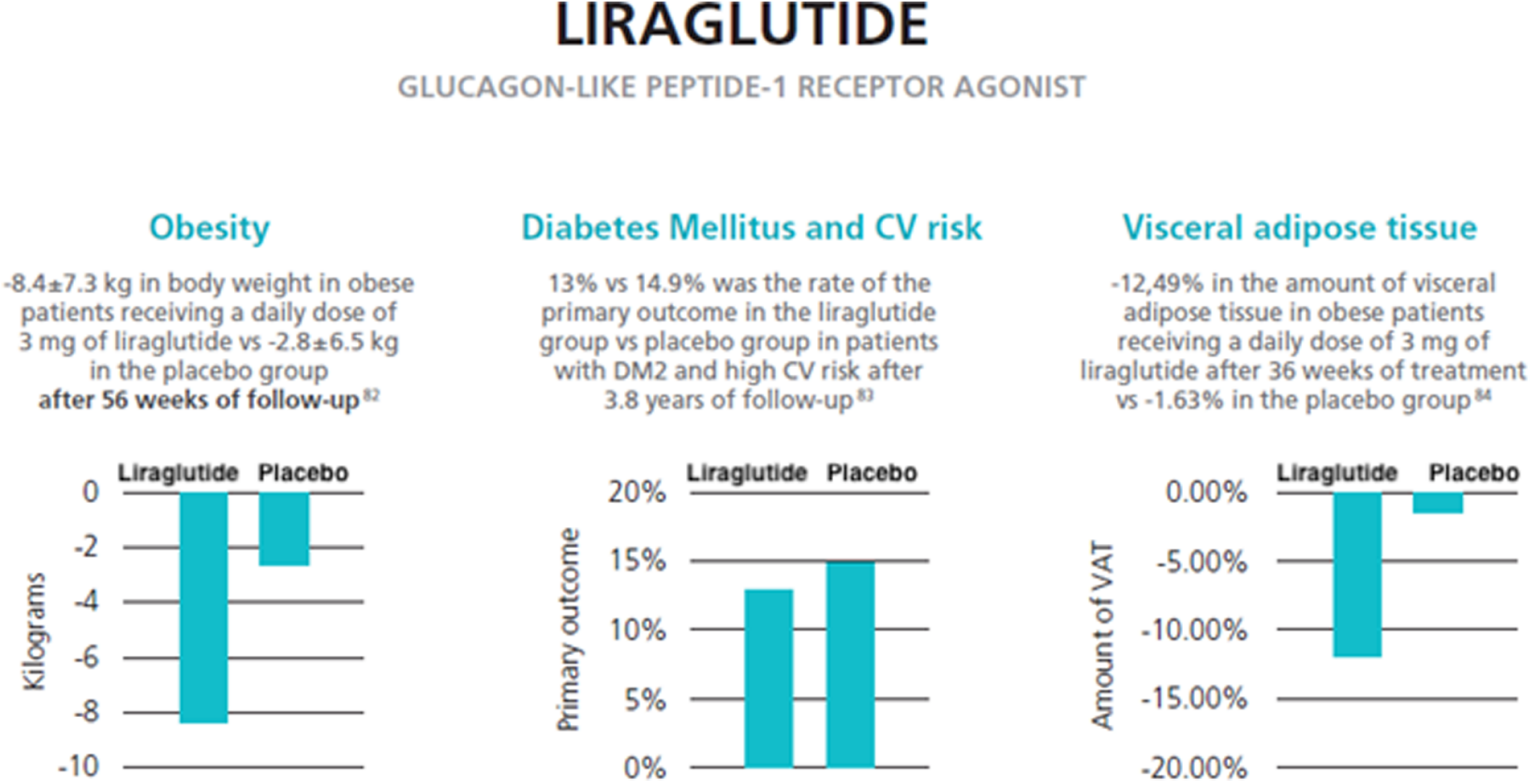
Effects of liraglutide on visceral adipose tissue and weight reduction (data from three reviews) (121–123).

The effects of liraglutide on visceral adipose tissue reduction are summarized in Figure 2.

## Drug interactions of the main weight-loss dietary supplements

The use of dietary supplements promoted for weight management is very common among consumers, attracted by promises on thermogenic properties (e.g., caffeine, green tea extracts), effects on satiety (e.g., glucomannan), increased fat oxidation and energy expenditure (e.g., pepper) (124).

Dietary weight-loss supplements might interact with prescription drugs, over-the-counter medications and herbal compounds. The potential for possible interactions should always be considered (125).

These interactions can be of pharmacokinetic or pharmacodynamic nature, or both. At pharmacokinetic level, interferences with hepatic cytochrome P450 (CYP450) enzymes are typical of St. John's wort or bitter orange, used for promoting a sense of satiety or increasing energy expenditure, respectively (125). Futhermore many supplements may interfere with absorption of drugs, such as laxatives (senna) (124). At pharmacodynamic level, alteration of dose–response relationship of insulin has been well described for supplements containing caffeine (124).

New natural constituents with anti-obesity effects are continuously being proposed, e.g., Vaccinium corymbosum, Vaccinium myrtillus, Tripterygium wilfordi (celastrol), astaxanthin, artemisinin, Ananas comosus, papain, saffron, tocopherol, etc. (126).

Few interactions are described between herbal compounds and approved anti-obesity drugs, such as orlistat, phentermine/topiramate, naltrexone/bupropion, liraglutide, semaglutide, and setmelanotide (127). Pharmacological interactions are more studied for older compounds, but this doesn't mean that interactions with herbs won't be recognized in the future for new treatments.

Orlistat reduces the absorption of beta-carotene and vitamin E when using for long-term. Other fat soluble vitamins (A, D, and K) may show small reductions in circulating concentrations, so that multivitamin supplements are recommended (128). Interestingly, psyllium natural fibers are helpful in controlling the gastrointestinal side effects of orlistat, reduced significantly in intensity after 60 days of supplementation (129).

There is a warning about the use with caution of St. John's wort with lorcaserin (10 mg) (130).

No data supporting herb-drug interactions are available specifically related to use of topiramate at low doses as an anorectic agent (125). Caffeine should be avoided or used cautiously with phentermine and some, but not all, green tea products contain caffeine (131).

Naltrexone has been shown to interact with several cytochrome P450 enzymes, particularly 2C9 and 2D6, in preclinical studies. However, the clinical significance of these interactions is not known (132).

Although Gimko biloba extract treatment appears to reduce significantly the half-life and increase the maximal plasmatic concentration of hydroxybupropion, no bupropion dose adjustments appear warranted when the drug is administered orally with G. biloba extract (133).

Interactions of liraglutide or semaglutide with herbal products have not been established (113, 134). Liraglutide has shown very low potential to be involved in pharmacokinetic drug-drug interactions related to cytochrome P450 and plasma protein binding (135).

Setmelanotide did not appear to be metabolized by human hepatic microsomes (X35).

## Conclusion

The main visceral adipose tissue is fatty tissue around the abdominal visceral organs. Waist circumference (WC) is the best anthropometric measure of VAT; CT and MRI are the most precise diagnostic techniques but are not always available. Several studies have shown a positive relationship between the size of VAT and CV disease. Indeed, VAT favors the development of atherogenic dyslipidemia, insulin resistance, and a low-grade systemic inflammatory state. Among anti-obesity drugs, a GLP-1 agonist called liraglutide (a subcutaneous antidiabetic drug), in addition to diet and physical exercise, demonstrated the ability to obtain a significant decrease in the size of VAT in a population of obese patients without diabetes mellitus. This positive result was also enhanced by reducing the blood level of CRP in patients receiving liraglutide. Similar results came from a recent trial of obese patients with DM.
